# Effects of coping on nurses' mental health during the COVID‐19 pandemic: Mediating role of social support and psychological resilience

**DOI:** 10.1002/nop2.1709

**Published:** 2023-03-22

**Authors:** Ying Xu, Qing‐Xiang Zheng, Xiu‐Min Jiang, Sheng‐Bin Guo, Yu‐Lan Kang, Yu‐Ping Lin, Gui‐Hua Liu

**Affiliations:** ^1^ Fujian Maternity and Child Health Hospital College of Clinical Medicine for Obstetrics & Gynecology and Pediatrics, Fujian Medical University Fuzhou City China; ^2^ Fujian Obstetrics and Gynecology Hospital College of Clinical Medicine for Obstetrics & Gynecology and Pediatrics, Fujian Medical University Fuzhou City China

**Keywords:** COVID‐19, mental health, nurse, structural equation model

## Abstract

**Background:**

Fighting against the COVID‐19 pandemic, front‐line nurses were under unprecedented psychological pressure. Therefore, it is necessary to promptly evaluate the psychological status of nurses during the COVID‐19 epidemic period.

**Aim:**

To investigate nurses' mental health during the COVID‐19 pandemic, and to test the mediating role of social support and psychological resilience between coping and mental health.

**Design:**

This was a descriptive, cross‐sectional survey which used a structural equation model.

**Method:**

In total, 711 registered nurses were included. All participants were invited to complete a socio‐demographic questionnaire, the general health questionnaire, the trait coping style questionnaire, the perceived social support scale and the Conner–Davidson Resilience scale.

**Results:**

In total, 50.1% nurses had high risk of mental health. Positive coping positively affected social support and psychological resilience, while it negatively affected mental health. Negative coping negatively affected social support and psychological resilience, while it positively affected mental health. Social support positively affected psychological resilience, while it negatively affected mental health. In addition, social support mediated coping and psychological resilience, and coping and mental health. Moreover, psychological resilience negatively affected mental health, and it mediated coping and mental health.

## 1 INTRODUCTION

The nurses' mental health is an important issue (Jung et al., 2021). However, nurses' mental health problems are also very common due to the fact that nurses are confronted with numerous sources of stress, such as shift work, long time work, sleep problems, psychological problems, a low tolerance for error and high patients' expectations for nurses (Mosadeghrad, 2013; Najimi et al., 2012; World Health Organization, 2022). The nurses' mental health was associated with decline in medical quality, patients' satisfaction and patients' safety (Chen et al., 2020; Gong et al., 2014; Lin et al., 2016). Under the influence of COVID‐19 pandemic, clinical nurses are under unprecedented physical fatigue and psychological distress, which lead to nurses further suffering the symptoms of psychological disorders like anxiety and depression, and sleep disturbance (Nie et al., 2020). In order to prevent decline in service quality, nurse mangers should evaluate the prevalence of psychological disorders among nurses (Jamali & Ayatollahi, 2015), especially during the COVID‐19 epidemic period.

Coping style, social support and psychological resilience are of great significance to nurses' mental health (Chen et al., 2020; Guo et al., 2017). Coping is ‘a process of adjustment following an adverse event’ (Rees et al., 2015). Compared with negative coping, positive coping seemed to have good adjustment to chronically stressful events (Lin et al., 2010). Coping affects psychological functioning via the mediating effects of psychological resilience (Rees et al., 2014). Psychological resilience is a very important personal quality to having good adaption to face to stress, dilemma, adversity and trauma (White et al., 2008), and enables nurses to actively adapt to job challenges (Delgadoa et al., 2017). Social support were regarded as protective factors against mental health (Feng et al., 2018). Individuals with greater support from their social networks tend to experience fewer psychological and physical health problems (Lin et al., 2010).

We found that even though several studies have found the single effect of coping, social support and psychological resilience on mental health, there are few studies that have analysed these variables in the same model or theoretical framework, and then studied their interacting effect on nurses' mental health. Therefore, it is essential for nursing managers to analyse key intermediary factors resulting in nurses' mental health, so as to take effective preventive intervention to decrease nurses' psychological disorders.

## 2 BACKGROUND

The COVID‐19 pandemic brought severe impacts on global health systems (World Health Organization, 2021). Fighting against the COVID‐19 pandemic and working directly with COVID‐19 patients, front‐line nurses were under unprecedented psychological pressure (Nie et al., 2020). Especially, they tend to develop mental health and psychological issues (Labrague, 2021). Evidence showed that 73.8% of front‐line nurses felt subject to stress resulting from the COVID‐19 according to the IES‐R score, which had higher impacts than the SARS epidemic (Nie et al., 2020; Wu et al., 2009). This could further lead to increasing workload, high turnover rates and nursing shortage. Nursing shortage has become an urgent concern worldwide (Li et al., 2016). Before the COVID‐19 pandemic, there were nearly 6 million nurses shortage worldwide (Shaffer et al., 2020). After the COVID‐19 outbreak, the requirements of nursing workforce has dramatically increased (Fan et al., 2021), which mainly caused the increase of nurses turnover intention rate (Labrague & de Los Santos, 2021). In addition, there will be a shortage of 18 million health care staff by 2030 worldwide (World Health Organization, 2020). Therefore, it is necessary to promptly evaluate the psychological status of nurses during the COVID‐19 epidemic period, and to address the negative impacts of the COVID‐19 pandemic.

### 2.1 Aims and hypothetical model

#### 2.1.1 Aims

This study aimed to investigate nurses' mental health during the outbreak of COVID‐19, and to test the hypothetical model to estimate the effects of coping style, social support and psychological resilience on nurses' psychological status.

#### 2.1.2 Hypothetical model

**FIGURE 1:**
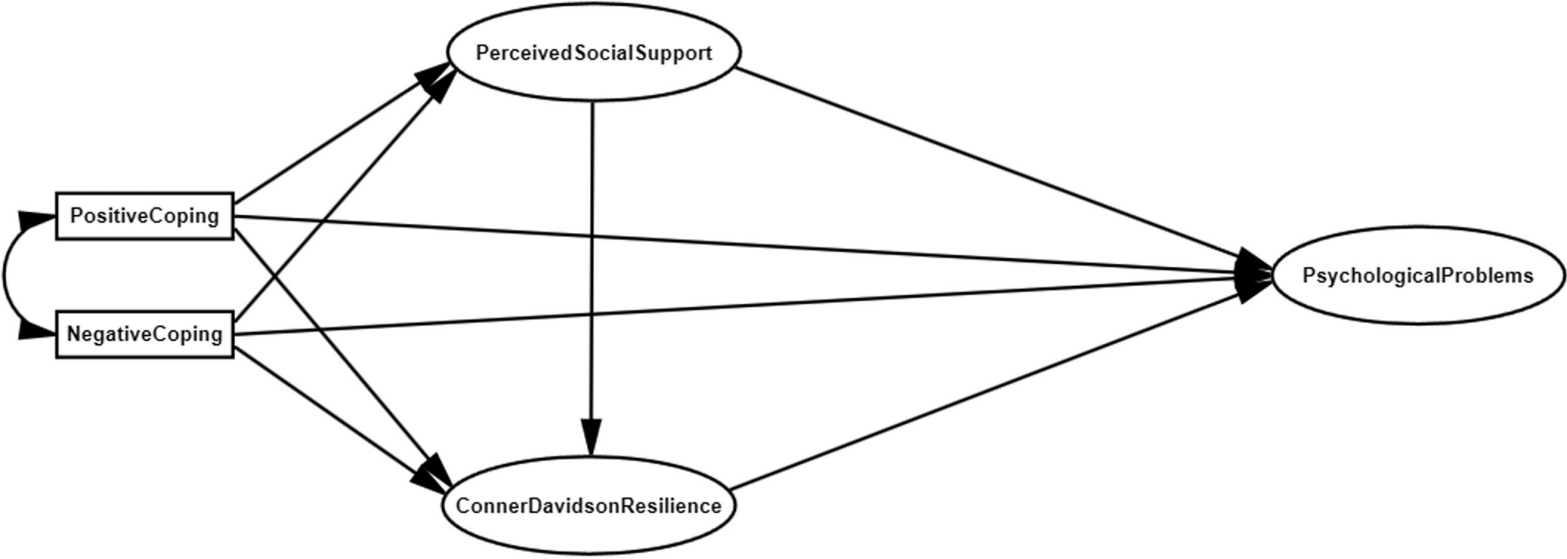
Research framework and hypotheses.

This study was constructed following eight hypotheses based on the above literature (Figure 1).

Hypothesis 1 Positive coping has significant effects on social support, psychological resilience and mental health.

Hypothesis 2 Negative coping also has significant effects on social support, psychological resilience and mental health.

Hypothesis 3 Social support has a significant effect on psychological resilience.

Hypothesis 4 Social support has a significant effect on mental health.

Hypothesis 5 Social support also can mediate mental health.

Hypothesis 6 Psychological resilience has a significant impact on mental health.

Hypothesis 7 Psychological resilience can mediate mental health.

## 3 METHODS

### 3.1 Design

We designed this study as a cross‐sectional and multicentre survey to test our hypothesized model. Meanwhile, we also followed the Strengthening the Reporting of Observational Studies in Epidemiology (STROBE) checklist (see Appendix S1).

### 3.2 Participants and sample size

The participants were midwives, maternity nurse, paediatric nurses and gynaecology nurses who were working at maternal and children hospital in China (REDACTED). The sample size of this study was estimated according to the minimum numbers of sample size to perform a structural equation model (Tinsley & Tinsley, 1987). More than 700 nurses at maternal and children hospital in Fujian province, China, were selected using cluster sampling, which was sufficient for structural equation model analysis. The inclusion criteria of this study were as follows: (1) nurse with a Registered Nurse license in China; (2) nurse with more than 1 year work experience, and (3) nurse who was volunteering for the study. Nurses who were on annual leave, sick leave and maternity leave during the investigation period were excluded.

### 3.3 Data collection

The data were collected using questionnaires by an online platform named WenJuanXing during September 2020 to October 2020. We set requirements in this platform for keeping data complete. The requirements were that each question must be answered, and each participant could get only one chance to submit their answers to platform. Before investigation, the research team trained charge nurses as the clinical investigators. They clearly explained the purpose of this study to all potentially eligible nurses. All participants were informed the details of study aims and their rights to decline or withdraw from this questionnaire survey at any time. In addition, they also informed that all data will be protected by the research group and will use it for academic purpose. Then, all participants were required to complete the questionnaires.

### 3.4 The questionnaires

The questionnaires of this study comprised of five parts. The socio‐demographic questionnaire included nurses age, institution, department, job title, position, education level, marital status, fertility status, employment type, work year, and frequency of night shift.

#### 3.4.1 General health questionnaire

The General Health Questionnaire (GHQ‐12) is the most well‐known and widely used questionnaire for detecting minor mental health due to its brevity, high sensitivity and specificity (Goldberg et al., 1997; Hystad & Johnsen, 2020). The GHQ‐12 consisted of 12 items, including 6 forward questions and 6 reverse questions. According to the scoring standard of the WHO (Von Korff et al., 1995), the 0‐0‐1‐1 or 1‐1‐0‐0 scoring method was used in this scale. The score of each question was 0 or 1, and the total score ranged from 0 to 12 points. The lower the score, the lesser the possibility of psychological disorder and vice versa. Based on the optimal cut‐off value of the GHQ‐12, the subjects were divided into two groups: high‐risk situation (GHQ‐12 score ≥4 points) and low‐risk situation (GHQ‐12 score <4 points). The Cronbach's α of the GHQ‐12 was 0.790.

#### 3.4.2 Trait coping style questionnaire

The trait coping style questionnaire (TCSQ) is used to determine the coping style of subjects, with a total of 20 items, and uses five‐point Likert scoring method (Xie, 1998). This scale included two dimensions: positive coping and negative coping (10 items each). The higher score of positive coping indicates that the more possibility of positive coping would be chosen. The higher score of negative coping indicates that the more possibility of negative coping would be chosen. The score of positive coping >40 meant active positive coping, while the score of negative coping >35 indicated obviously negative coping would be chosen. The Cronbach's α of the TCSQ was 0.934 in this study.

#### 3.4.3 Perceived social support scale

The perceived social support scale (PSSS) is a tool to measure personal self‐perception and multilevel social support (Blumenthal et al., 1987; Jiang, 2001). The PSSS comprised of 12 items and 3 dimensions including family support (4 items), friend support (4 items) and other support (4 items). This scale uses the 7‐point Likert scoring method, and its total score ranged from 12 to 84 points. The higher total score indicates the higher level of perceived social support. The score between 12 and 36 points indicates low support level; between 37 and 60 points indicates intermediate support level, and between 61 and 84 points indicates high support level. The Cronbach's α coefficient of the PSSS in this study was 0.934. Besides, the test–retest reliability of the PSSS was 0.85, and the test–retest reliability of family support, friend support and other support was 0.85, 0.75 and 0.72, respectively.

#### 3.4.4 The Chinese version of Conner–Davidson resilience scale

The Conner–Davidson Resilience scale (CD‐RISC) is the most widely used psychological resilience scale (Connor & Davidson, 2003; Yu et al., 2011), with a total of 25 items and 3 dimensions including tenacity dimension (13 items), strength dimension (8 items) and optimism dimension (4 items). This scale uses the five‐point Likert scoring method, and its total score ranged from 0 to 125 points. The total score of 0–56 points indicates a lower level of psychological resilience, a total score of 57–70 points indicates a medium level of psychological resilience and a total score of more than 71 points indicates a higher level of psychological resilience. The higher score indicates the higher level of psychological resilience of the subjects. The reliability Cronbach alpha of the CD‐RISC was 0.91 in this study.

### 3.5 Ethics statement

This study was approved by hospital's Institutional Review Board before formal survey (Approval number: 2020YJ224).

### 3.6 Data analysis

The SPSS software (version 25.0) were used to analyse the data. The demographic characteristics and research variables were described using means, standard deviations (SDs) and percentages. The relationships among variables were tested using Spearman's correlation analysis and logistic regression analysis. The AMOS software (version 26.0) was used for structural equation modelling (SEM) and path analysis, and the maximum likelihood method was used to estimate the hypothetical model. The fitness of this model was evaluated using the following indices: *χ* ^2^/*df*, AGFI, GFI, TLI, IFI, NFI, CFI and RMSEA. The criteria of the adequate model required indices to accord with the recommended threshold values: *χ* ^2^/*df* >1.00 and <3.00, TLI >0.90, IFI >0.90, NFI >0.90, CFI >0.90 and RMSEA <0.08 (Wu, 2013). In addition, the Bootstrap test (5000 bootstrapping repetitions) was performed to test the significance of the model's indirect and total effects. The *p* < 0.05 suggested statistically significant.

## 4 RESULTS

### 4.1 Characteristics of the participants

A total of 711 questionnaires were distributed in this study, of which 709 were valid, and the effective recovery rate was 99.72%. Two questionnaires were suspected of unreal answers due to each question of them were answered and selected the same answer. The average age of nurses was 31.35 (7.84) years, and 62.2% of them were married. Mostly participants were ‘Nurse’ and ‘Nurse Practitioner’ (81.5%), and 96.1% of them were in the position of nurse. More than half of nurses were undergraduate and above undergraduate (52.5%). All results are shown in Table 1.

**TABLE 1:** Sociodemographic characteristics of nurses (*N* = 709).

Variables	Categories	Mean (*SD*)/*N* (%)
Age (years)		31.35 (7.84)
	≤25	175 (24.7)
	26–35	360 (50.8)
	36–44	129 (18.2)
	≥45	45 (6.3)
Department	Obstetrics Department	112 (15.8)
	Delivery Room	21 (3)
	Gynecology Department	121 (17.1)
	ICU	24 (3.4)
	NICU, PICU, CCU	171 (24.1)
	Paediatric Internal Medicine Department	63 (8.9)
	Paediatric Surgery Department	47 (6.6)
	Operating Room	60 (8.5)
	Emergency department	15 (2.1)
	Outpatient	75 (10.6)
Job title	Nurse	224 (31.6)
	Nurse practitioner	354 (49.9)
	Nurse‐in‐charge	104 (14.7)
	Associate professor of nursing and above	27 (3.8)
Position	Nurse	681 (96.1)
	Head nurse or above	28 (3.9)
Education level	Junior college and below	337 (47.5)
	Undergraduate and above	372 (52.5)
Marital status	Married	441 (62.2)
	Single or divorced	268 (37.8)
Fertility status	Infertile	312 (44)
	One	253 (35.7)
	Two or more	144 (20.3)
Employment type	Contract employee	359 (50.6)
	Regular employee	350 (49.4)
Work year (year)	≤5	287 (40.5)
	5 < years ≤ 10	177 (25)
	10 < years < 20	152 (21.4)
	≥20	93 (13.1)
The frequency of night shift peer month (times)	8 < 20	306 (43.2)
	4 ≤ *n* < 8	72 (10.2)
	≥8	331 (46.7)

### 4.2 Means, SD and correlations of variables

The means, SD and correlations of variables are shown in Table 2. The statistical average score of the GHQ‐12 was 3.46 (2.52). Among the participants surveyed, 50.1% (355 of 709) nurses had high risk of mental health. Besides, the scores of positive coping [36.35 (6.06)] was higher than that of negative coping [29.10 (7.72)]. Meanwhile, the total score of the PSSS was 69.93 (11.22); the scores of family support, friend support and other support dimensions were 5.90 (1.03), 5.85 (0.96) and 5.72 (1.03), respectively. Moreover, the total score of the CD‐RISC was 66.36 (16.77). Among that, the score of the strength dimension was the highest [2.83 (0.69)] and that of the tenacity dimension was the lowest [2.60 (0.70)].

**TABLE 2:** Means, SD and correlations among variables (*N* = 709).

Variables	Mean (*SD*)	1	2	3	4	5	6	7	8	9	10	11	12
1. GHQ‐12	3.46 (2.52)	1.00											
2. Social support	69.93 (11.22)	−0.465**	1.00										
3. Family support	23.64 (4.12)	−0.420**	0.913**	1.00									
4. Friend support	23.41 (3.85)	−0.431**	0.929**	0.793**	1.00								
5. Other support	22.89 (4.13)	−0.441**	0.938**	0.780**	0.841**	1.00							
6. Coping		0.201**	0.109**	0.090*	0.112**	0.116**	1.00						
7. Positive coping	36.35 (6.06)	−0.475**	0.624**	0.570**	0.587**	0.598**	0.427**	1.00					
8. Negative coping	29.10 (7.72)	0.597**	−0.335**	−0.325**	−0.302**	−0.308**	0.679**	−0.285**	1.00				
9. Psychological resilience	66.36 (16.77)	−0.548**	0.658**	0.610**	0.620**	0.625**	0.120**	0.728**	−0.404**	1.00			
10. Tenacity dimension	33.35 (9.14)	−0.524**	0.620**	0.576**	0.585**	0.588**	0.108**	0.692**	−0.386**	0.979**	1.00		
11. Strength dimension	22.60 (5.52)	−0.551**	0.660**	0.606**	0.625**	0.631**	0.092*	0.720**	−0.426**	0.961**	0.907**	1.00	
12. Optimistic dimension	10.41 (2.79)	−0.474**	0.602**	0.556**	0.570**	0.567**	0.180**	0.678**	−0.316**	0.870**	0.804**	0.805**	1.00

**p < 0.01. *p < 0.05.

The correlation analysis showed that mental health was negatively correlated with social support, family support, friend support, other support, coping, positive coping, negative coping, psychological resilience, tenacity dimension, strength dimension and optimistic dimension (*p* < 0.05), while it was positively correlated with negative coping (*p* < 0.05). Besides, social support was positively associated with coping and psychological resilience, and coping was positively correlated with psychological resilience (*p* < 0.05).

### 4.3 Analysis of the GHQ‐12 each item

The results of the analysis of the GHQ‐12 each item showed that there were 63.8% (452 of 709) nurses who were ‘Lose sleep over worry’ in the past 1 month, 63.5% (450 of 709) nurses who were recently ‘constantly under strain’ (Table 3). Also, 56.3% (399 of 709) nurses felt ‘Unhappy or depressed’, and 54.2% (384 of 709) nurses could not face up to problems lately.

**TABLE 3:** Analysis of the GHQ‐12 each item.

GHQ‐12 item	Number of Nurse (*N*)	Rate (%)
1. Unable to concentrate	16	2.3
2. Lose sleep over worry	452	63.8
3. Think of self as worthless	36	5.1
4. Incapable of making decisions	29	4.1
5. Constantly under strain	450	63.5
6. Could not face up to problems	384	54.2
7. Could not enjoy day‐to‐day activities	61	8.6
8. Could not overcome difficulties	54	7.6
9. Unhappy or depressed	399	56.3
10. Lose confidence in self	331	46.7
11. Play useless part in things	196	27.6
12. Feel reasonably unhappy	42	5.9

### 4.4 Logistic regression analysis of influencing factors for nurses' mental health

The influencing factors for nurses' mental health, including age, department, job title, position, education level, marital status, fertility status, employment type, work year, the frequency of night shift peer month, coping, social support and psychological resilience, were analysed by the logistic regression analysis (the Wald stepwise regression method). After adjusted analysis, coping, social support and psychological resilience were influencing factors for nurses' mental health (*p* < 0.05, Table 4). The results of logistic regression analysis indicated that nurses who with higher coping score (odds ratio (OR) = 1.097, 95% confidence interval (95% CI): 1.072, 1.124, *p* < 0.0001) were associated with poor mental health during the COVID‐19 pandemic; conversely, nurses with higher level of social support (OR = 0.936, 95% CI: 0.922, 0.951, *p* < 0.0001) and psychological resilience (OR = 0.951, 95% CI: 0.931, 0.972, *p* < 0.0001) were related with better mental health.

**TABLE 4:** Logistic regression analysis results of influencing factors for nurses' mental health.

Variable	B	SE	Wald value	*p*	OR	95% CI of OR	
						Lower limits	Upper limits
Coping	0.093	0.012	59.91	<0.0001	1.097	1.072	1.124
Social Support	−0.066	0.008	70.93	<0.0001	0.936	0.922	0.951
Psychological resilience	−0.05	0.011	20.82	<0.0001	0.951	0.931	0.972
Constant	1.709	0.85	4.037	0.045	5.522	/	/

### 4.5 Fitness of the hypothetical model

**FIGURE 2:**
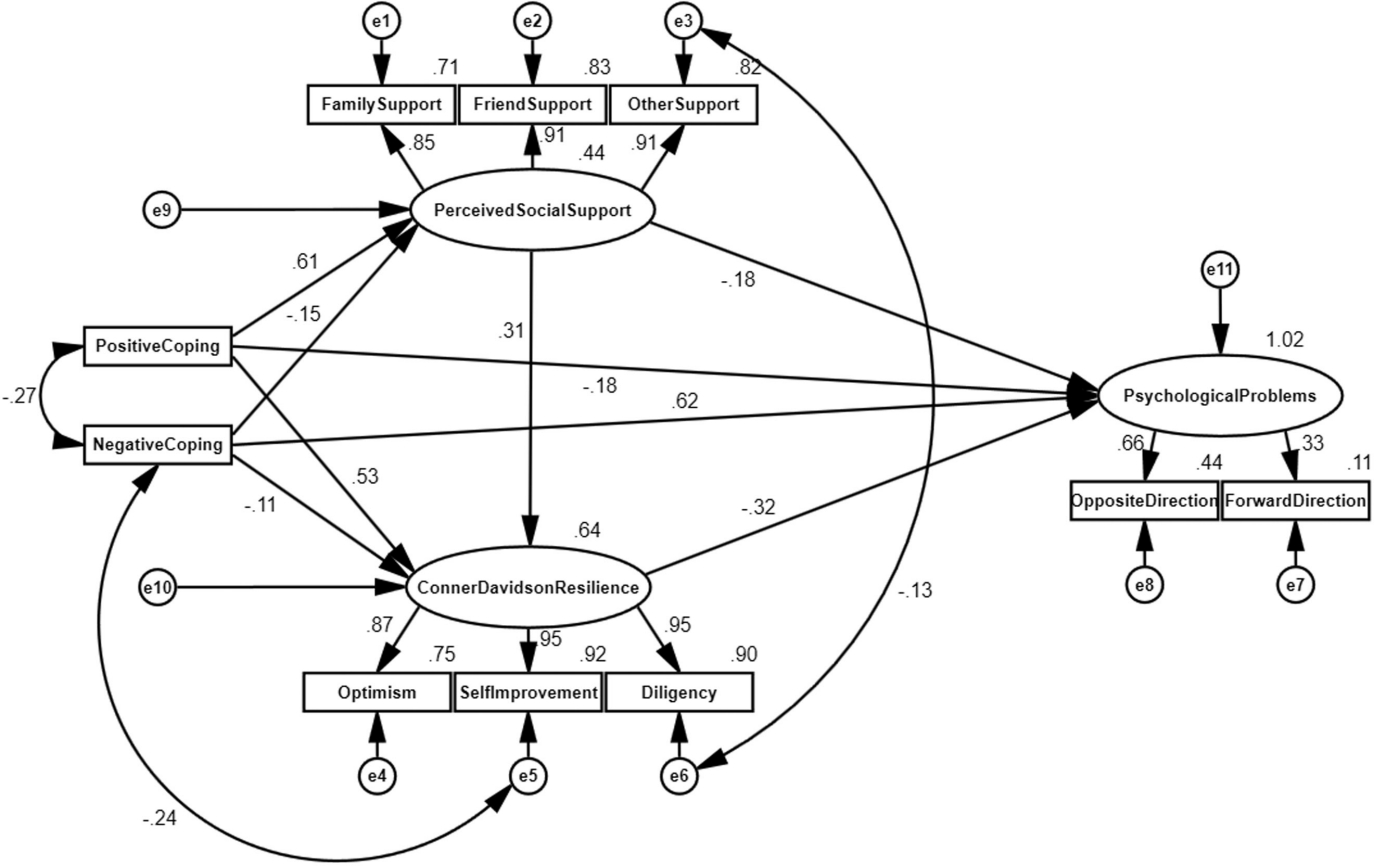
The final research model with standardized path coefficients.

Based on the hypothetical model, the structural equation model was established to verify the relationships among coping, social support, psychological resilience and mental health (Figure 2). The results of fitting model indexes indicated that the model fitted satisfactorily to the data: χ^2^/df = 1.989, RMSEA = 0.037, CFI = 0.997, IFI = 0.993, TLI = 0.997, and NFI = 0.993. In addition, the whole model explained 29% of the variance in social support, 29% of the variance in psychological resilience and 18% of the variance in mental health.

The results of model analysis showed that positive coping had a direct positive effect on mental health, and it also had indirect effects on mental health via social support and psychological resilience (*p* < 0.05), which supported Hypothesis 1. Meanwhile, negative coping had a direct negative effect on mental health, and it also had indirect effect on mental health through social support and psychological resilience (*p* < 0.01), which supported Hypothesis 2. Besides, Hypothesis 3 was also supported. Social support had a direct positive effect on psychological resilience (*p* < 0.01). Hypotheses 4 and 5 were supported in this model. It was found that the direct, indirect and mediated effects of social support on mental health were significant (*p* < 0.05). Furthermore, the direct and mediated effects of psychological resilience on mental health were significant (*p* < 0.01), which supported Hypotheses 6 and 7. All results are shown in Table 5.

**TABLE 5:** Total effects, direct effects and indirect effects of each path in this model.

Estimate	β	BC 95% CI^a^		*p*
		Lower limits	Upper limits	
Total effects				
Positive Coping → Social Support	0.606	0.552	0.658	<0.001
Positive Coping → Psychological resilience	0.730	0.682	0.773	<0.001
Positive Coping → Mental health	−0.360	−0.417	−0.299	<0.001
Negative Coping → Social Support	−0.150	−0.201	−0.097	<0.001
Negative Coping → Psychological resilience	−0.155	−0.224	−0.094	<0.001
Negative Coping → Mental health	0.470	0.418	0.521	<0.001
Social Support → Psychological resilience	0.320	0.245	0.391	<0.001
Social Support → Mental health	−0.199	−0.285	−0.111	<0.001
Psychological resilience → Mental health	−0.223	−0.330	−0.113	<0.001
Direct effects				
Positive Coping → Social Support	0.606	0.552	0.658	<0.001
Positive Coping → Psychological resilience	0.536	0.471	0.604	<0.001
Positive Coping → Mental health	−0.120	−0.212	−0.026	0.012
Negative Coping → Social Support	−0.150	−0.201	−0.097	<0.001
Negative Coping → Psychological resilience	−0.107	−0.174	−0.048	<0.001
Negative Coping → Mental health	0.416	0.359	0.471	<0.001
Social Support → Psychological resilience	0.320	0.245	0.391	<0.001
Social Support → Mental health	−0.127	−0.220	−0.037	0.006
Psychological resilience → Mental health	−0.223	−0.330	−0.113	<0.001
Indirect effects				
Positive Coping → Psychological resilience → Mental health	0.194	0.148	0.243	<0.001
Positive Coping → Social Support→ Psychological resilience → Mental health	−0.240	−0.320	−0.162	<0.001
Negative Coping → Psychological resilience → Mental health	−0.048	−0.071	−0.028	<0.001
Negative Coping → Social Support → Psychological resilience → Mental health	0.054	0.032	0.081	<0.001
Social Support → Psychological resilience → Mental health	−0.072	−0.113	−0.038	<0.001

^a^ Means that 95% bias‐corrected bootstrap confidence interval.

The total effect of positive coping to mental health was β = −0.360; among this, the direct effect was β = −0.120, accounting for 33.33% of the total effect, and the total indirect effect was β = −0.240, accounting for 66.67%. The total effect of negative coping to mental health was β = 0.470; among this, the direct effect was β = 0.416, accounting for 88.51% of total effect, and the total indirect effect was β = 0.054, accounting for 11.49%. In addition, the direct effect of social support to psychological resilience was β = 0.320. The total effect of social support to mental health was β = −0.199; among this, the direct effect was β = −0.127, accounting for 63.82% of total effect, and the total indirect effect was β = −0.072, accounting only for 36.18%. Besides, the direct effect of psychological resilience to mental health were β = −0.223. All results are shown in Table 5.

## 5 DISCUSSION

Clinical nurses, who were the most providers of health care system, were under unprecedented psychological fatigue (Nie et al., 2020). The results of this study further supported these negative impacts. In addition, the statistical average score of the GHQ‐12 in this study was higher than that of Iranian nurses (Jamali & Ayatollahi, 2015). This might be related to the fact that compared with Iranian nurses, nurses in this study had experienced a nervous atmosphere and overload working during the COVID‐19 epidemic. Besides, there were 63.8% nurses who were ‘Lose sleep over worry’ in the past 1 month and 63.5% nurses who were recently ‘Constantly under strain’ (Table 3). Also, 56.3% nurses felt ‘Unhappy or depressed’ and 54.2% nurses could not face up to problems lately. All of these results suggested that ahypnosis, pressure, depression and anxiety were the main psychological problems of nurses during the COVID‐19 epidemic. These findings were consistent with the findings of Rubin and Wessely (2020) that anxiety was likely to increase further during the COVID‐19 pandemic. In addition, health care workers closely contacting with the COVID‐19 should receive psychological crisis intervention (Jiang et al., 2020). Therefore, nursing managers should focus on nurses whether they suffered these psychological problems, and strengthen prevention and intervention. It is better for nursing managers to carry out psychological reaction evaluation for nurses in time so as to alleviate nurses' fears and doubts about the COVID‐19 epidemic.

Positive coping strategies could relieve stress (Lin et al., 2010). This study found that nurses were more likely to take positive coping during the COVID‐19 pandemic, which was consistent with previous studies (Isa et al., 2019; Nie et al., 2020; Zhou & Gong, 2015). Besides, the results of the structural equation model illustrated that the total effects of negative coping on mental health (β = 0.470) accounted to more impacts than that of positive coping (β = −0.360). Moreover, the negative coping tended to directly affect mental health (88.51%), while positive coping was more likely indirectly affect mental health (66.67%). Therefore, nursing managers can concentrate on nurses' negative behaviours and psychological pressure during the COVID‐19 pandemic, and take targeted intervention to improve coping styles and guide them to treat their daily work with a positive attitude, and then promote their mental health.

Social support was regarded as one of the important mediating factors that determine the relationship between psychological stress and health, which was the emotional experience of the individual feeling supported, respected and understood (Howard et al., 2017). The social support score of nurses in this study were 69.93 (11.22), higher than that reported by Liao et al. (2021). In addition, the average score of family support in this research was the highest, followed by the score of friend support and other support. This further suggested that social support from family were important protective factors for individual stress tolerance (Rueger et al., 2016). We also found that social support were negatively correlated with of nurses' mental health during the COVID‐19 epidemic, which was similar to the finding of Galanis et al. (2021) that decreased social support was one of the main risk factors to increase the burnout of nurses. This might be due to the fact that social support from colleagues and superiors could promote nurses' health and well‐being at work (Blanco‐Donoso et al., 2017; Chen et al., 2020). The higher the degree of social support was, the easier for nurses to solve problems and adapt to work pressure and difficulties from clinical working was, so as to avoid negative work results (Meng et al., 2017). Therefore, to promote their mental health, nursing managers should provide a supportive work environment for nurses, especially in the COVID‐19 pandemic period.

The results of this study provided evidence that individuals with higher psychological resilience usually adopt optimistic and active attitudes under stressful conditions, and they may know how to use external resources to handle problems, which were consistent with Xie and Fan (2014). This study also showed that psychological resilience score of nurses was 66.36 (16.77), which was higher than Pan et al.'s (2020) psychological resilience score of 64.26 (15.13). In addition, the score of optimism factor in this study was the lowest, while the score of tenacity factor was the highest. This might be due to the fact that nurses were nervously fighting with the COVID‐19 epidemic every moment, no matter what their positions were.

This study explained that coping, social support and psychological resilience were the influencing factors, which independently affected nurses' mental health, and the mediating effects of social support and psychological resilience between coping and mental health among nurses during the COVID‐19 pandemic for the first time. Meanwhile, the results of this model analysis showed that Hypotheses 1–7 were supported. Positive coping had a positively direct effect on mental health, and it also had negatively indirect effects on mental health via social support and psychological resilience. Also, negative coping had a negatively direct effect on mental health, and it also had positively indirect effects on mental health via social support and psychological resilience. Social support positively affected psychological resilience, while it had negative effect on mental health. It was illustrated that social support could mediate coping, psychological resilience and mental health. Psychological resilience negatively affected mental health, and it could mediate coping and mental health. Therefore, nursing managers can guide clinical nurses how to positively cope with tough work, enhancing their social support and psychological resilience to effectively promote their mental health during the COVID‐19 epidemic. Moreover, for mitigating the impact of the COVID‐19 pandemic on nurses' mental health, nursing managers also can take measurements like screening for the symptoms of mental health and psychological interventions for high‐risk nurses (Galanis et al., 2021). By this way, this might be to prevent or relieve nurses' psychological disorders, and then it might be improve the medical quality, patients' satisfaction and patients' safety.

### 5.1 Limitations

There were still several limitations which could inspire future directions of related research. First, this survey only performed in a maternity and child health hospital including two districts; however, the results of this study still had a certain reference value for other research. Second, this study was designed as a cross‐sectional study, and a longitudinal design study should be conducted in future to draw a certain conclusion. Third, there were several self‐reported questions in this survey, which might result in biases to results. Therefore, it is essential to consider multicentre and expanding randomly sample for further exploring the relationships among coping, social support, psychological resilience and nurses' mental health during public health epidemic, and validate the structural models.

## 6 CONCLUSIONS

Overall, coping, social support and psychological resilience were the influencing factors for nurses' mental health. In addition, social support and psychological resilience effectively mediated coping and mental health among nurses during the COVID‐19 pandemic. Further, positive coping, favourable social support and a higher level of psychological resilience had positively effects on nurses' mental health. Nursing managers can guide nurses how to positively cope with tough, enhance their social support and psychological resilience to effectively promote their mental health and take psychological interventions for high‐risk nurses.

## AUTHOR CONTRIBUTIONS

YX contributed to the data collection, statistical analysis and writing manuscript. QXZ contributed to the study design, statistical analysis, data interpretation, writing and revising manuscript. XMJ contributed to the study design, data interpretation and revising manuscript. SBG, YPL and YLK contributed to the study design and data interpretation. GHL contributed to the statistical analysis and data interpretation. All the authors contributed to the preparation of the manuscript and approved the final submitted version.

## FUNDING INFORMATION

None.

## CONFLICT OF INTEREST STATEMENT

The authors declare no conflict of interest.

## ETHICS STATEMENT

This study was approved by the Ethics Committee of Fujian Maternity and Child Health Hospital in China. All participants were informed about the aims and their consent to participate in this study was obtained. (Approval number: 2020YJ224).

## Supporting information


Appendix S1.

## Data Availability

Data sharing is not applicable to this article as no new data were created or analyzed in this study.
